# Finesse in Chondrolaryngoplasty

**DOI:** 10.1097/GOX.0000000000005539

**Published:** 2024-01-22

**Authors:** Jacqueline M. Ihnat, Kevin G. Hu, Mariana N. Almeida, Neil Parikh, Sacha C. Hauc, David P. Alper, Michael Alperovich

**Affiliations:** From the Yale School of Medicine, Department of Surgery, Plastic and Reconstructive Surgery, New Haven, Conn.

Chondrolaryngoplasty, or tracheal shave, is a procedure performed in facial feminization for gender-affirming facial surgery. The thyroid cartilage enlarges during puberty when exposed to elevated testosterone levels, creating the visible projection known as the laryngeal prominence or Adam’s apple.^1^ This stereotypically masculine feature can be a source of profound gender dysphoria for transfeminine patients, and its removal was initially described in 1975 by Wolfort and Parry.^2^ Advances in technique have since improved outcomes both aesthetically and functionally.^3^ Here, we present our approach to chondrolaryngoplasty with key modifications to optimize safety and aesthetic outcomes. (See Video [online], which displays our improved approach to chondrolaryngoplasty.)

**Video 1.** This video displays the authors’ approach to chondrolaryngoplasty.

Although a higher incision has been described, the procedure is most commonly described with an incision at the cervicomental junction.^4,5^ However, a prominent scar at the cervicomental junction can be a visible reminder of the patient’s surgical transition. We suggest placing an incision in the submental region, similar to the incision for a chin implant or neck tightening during a facelift. Prior research has identified scar appearance as the main source of dissatisfaction after chondrolaryngoplasty.^6^ By placing the scar in a more inconspicuous location, we have avoided the most common criticism of the procedure.

Our approach to the thyroid prominence is performed in the standard fashion. Although the distance between the incision and the thyroid prominence is longer, creating a potentially more difficult approach, a long-tipped electrocautery device allows for sufficient reach. Once the surgeon has tunneled under the fascia to the thyroid cartilage, the platysma and strap muscles are retracted away from the midline and the perichondrium is incised.

A needle is then inserted transcutaneously cranial to the anticipated location of the vocal card insertion. Direct flexible fiberoptic laryngoscopy is used to confirm positioning of the needle both before and after the procedure. Limiting cartilage excision to above the needle prevents destabilization of the vocal cords and epiglottis, minimizing risk to the patient’s voice.^4,7,8^ Through this method, we have successfully avoided vocal injury in 100% of cases.

Cartilage historically has been removed via rongeur, scalpel, electrocautery, or powered drill.^1,5^ The use of a diamond burr to better shape ossified cartilage has been applied in recent years,^9^ but can inadvertently damage surrounding soft tissue.

Our modification employs the use of an ultrasonic burr, which is atraumatic to soft tissue while providing a high degree of precision and remaining effective against calcified cartilage. The improved safety profile of the ultrasonic burr is especially useful when working from a more cranial incision that requires tunneling a longer distance to reach the target site with limited visibility.

Once the tracheal prominence has been adequately trimmed and the skin has been re-draped to ensure smooth contours, closure is performed in the typical fashion: strap muscles, platysma, and deep subcutaneous tissue are reapproximated before final closure of cutaneous fascia. Our finesse approach to chondrolaryngoplasty improves aesthetic outcomes through an incision in the submental area, allowing for better scar concealment. Additionally, the use of an ultrasonic burr for cartilage take down is efficient and prevents accidental damage to surrounding tissues.

## DISCLOSURES

Dr. Alperovich is a consultant for Johnson & Johnson and LifeNet Health. All the other authors have no financial interest to declare in relation to the content of this article.

## ACKNOWLEDGMENTS

This publication was made possible by CTSA Grant Number KL2 TR001862 from the National Center for Advancing Translational Science (NCATS), a component of the National Institutes of Health (NIH). Its contents are solely the responsibility of the authors and do not necessarily represent the official view of NIH.
